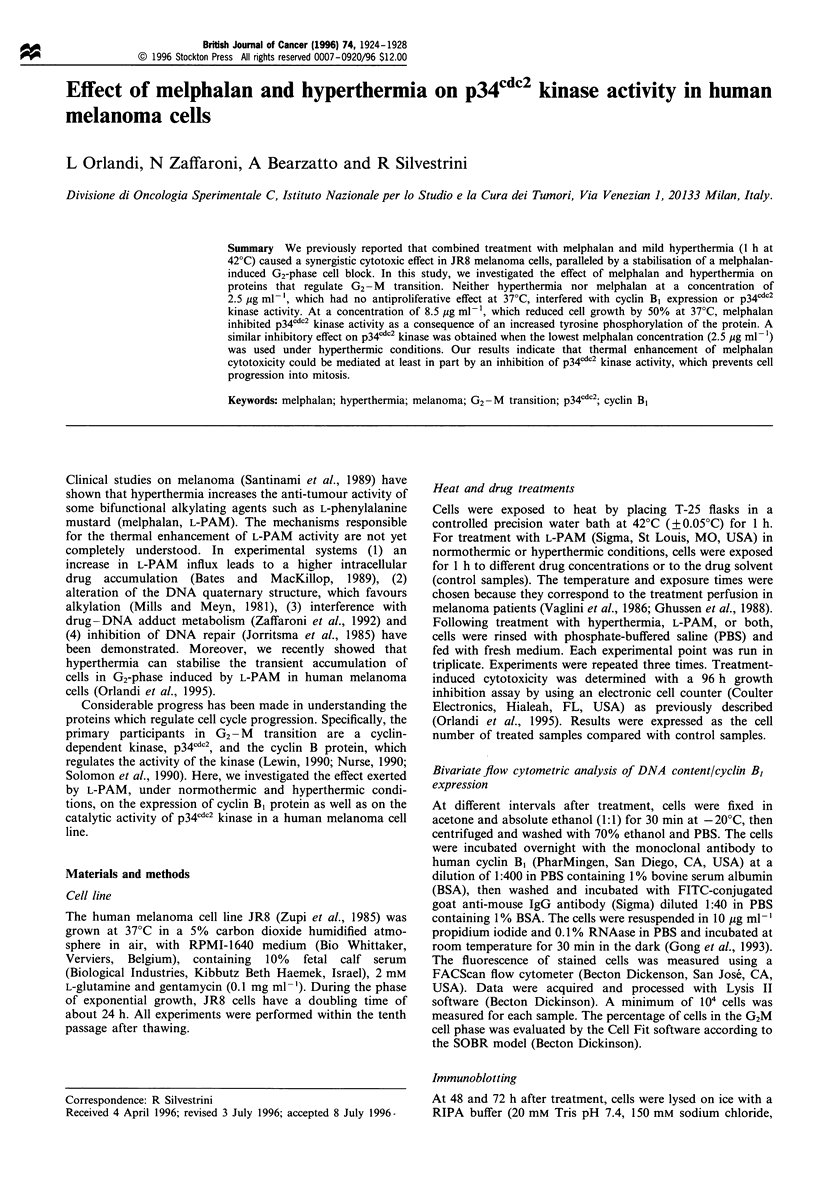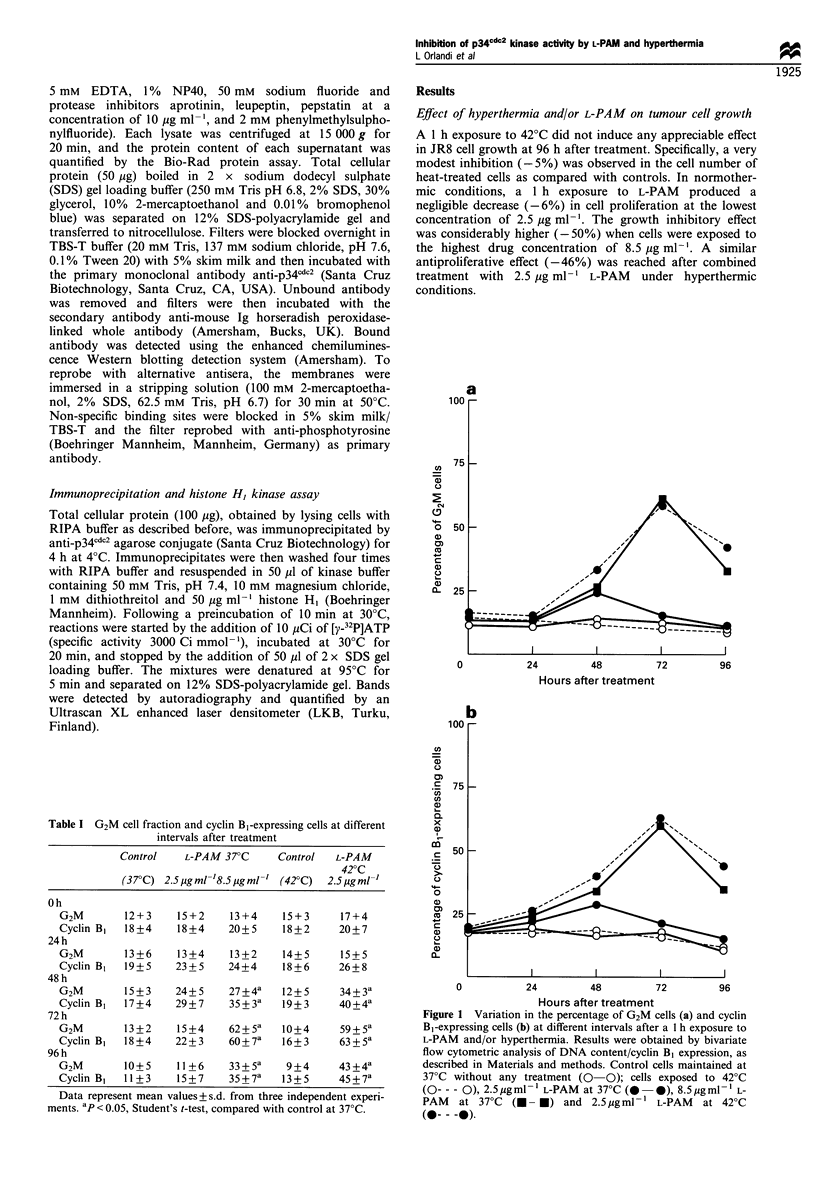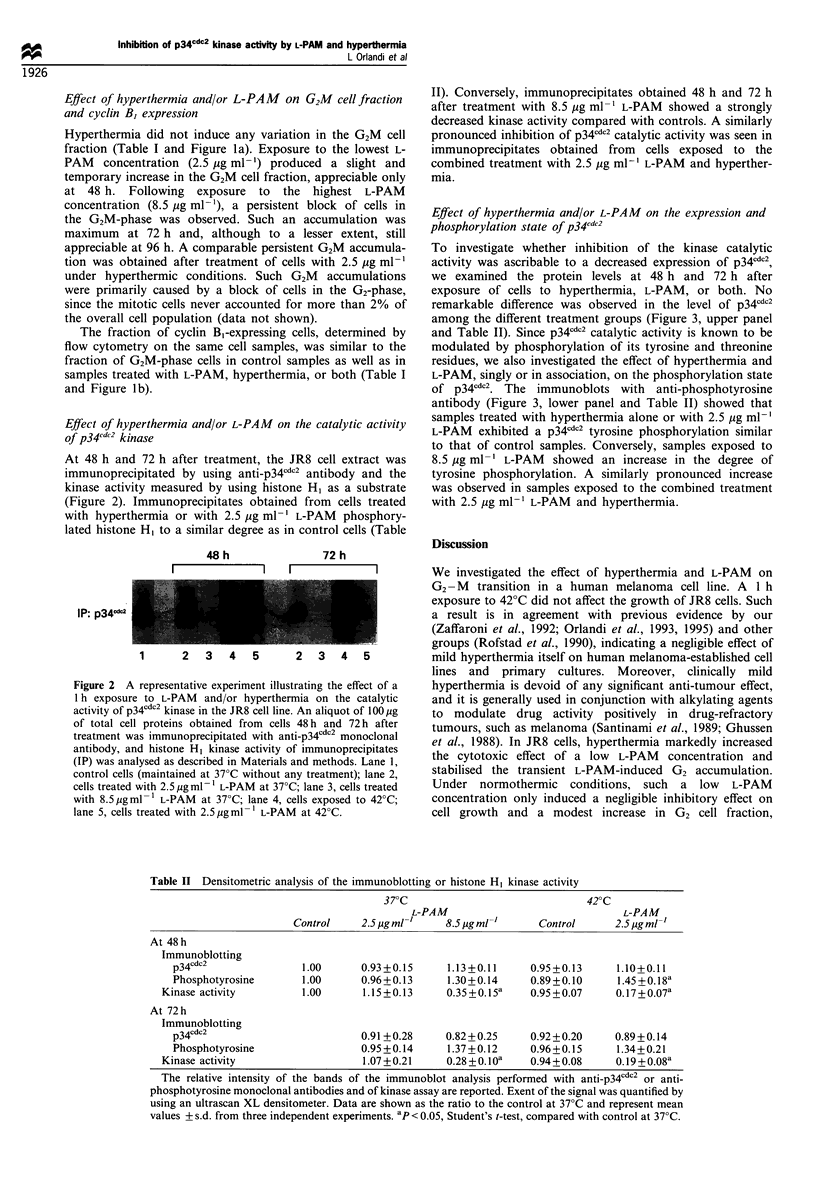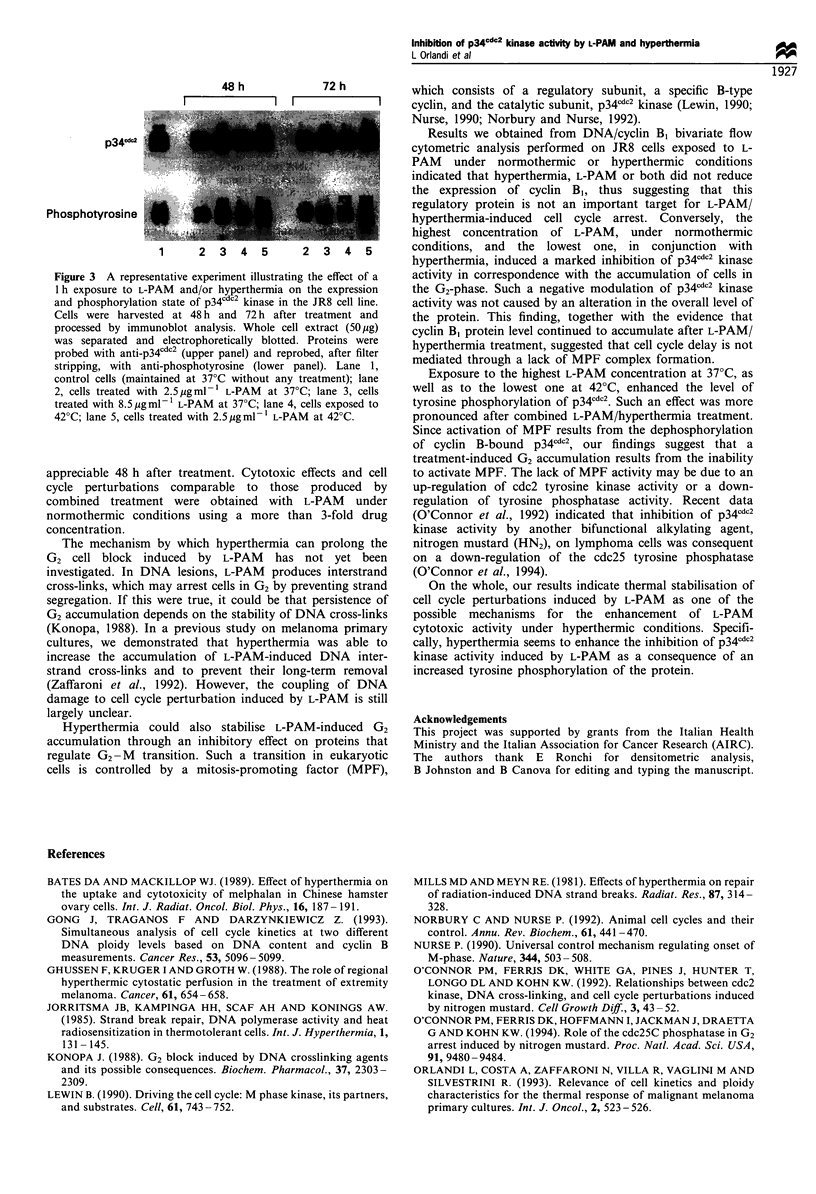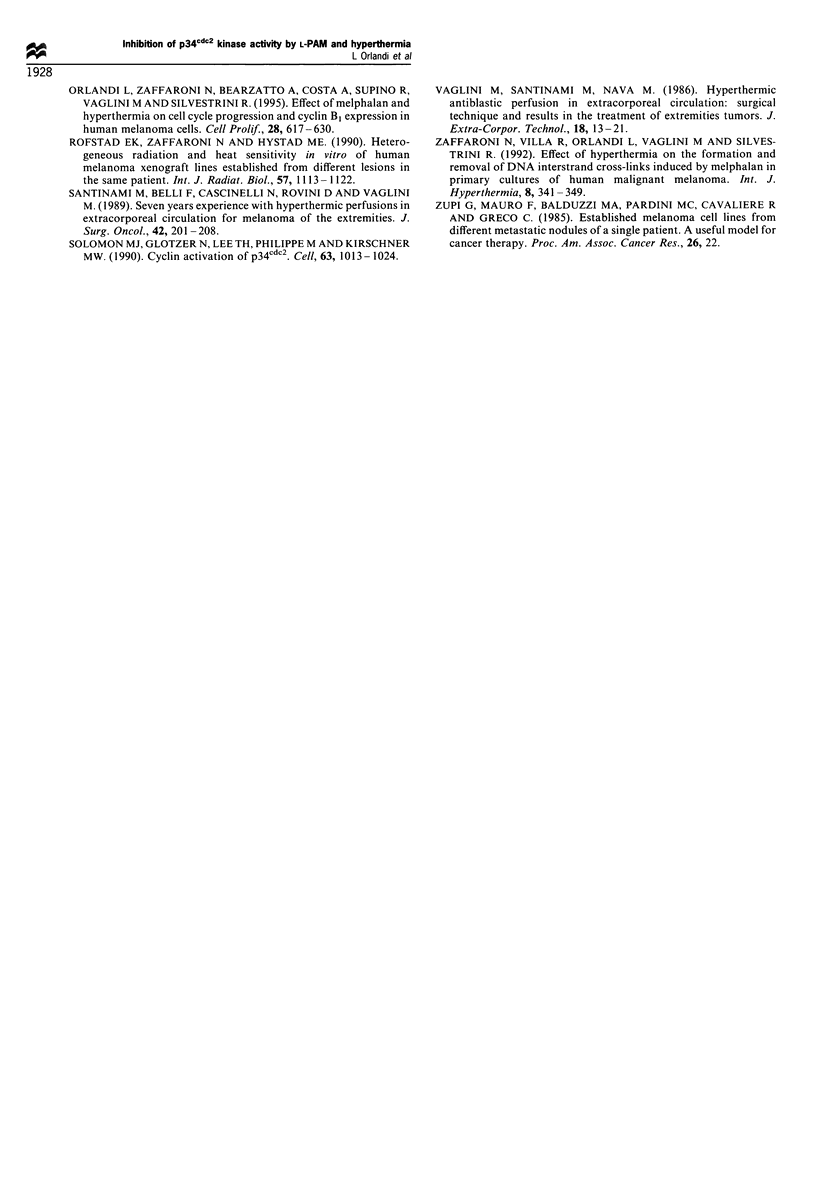# Effect of melphalan and hyperthermia on p34cdc2 kinase activity in human melanoma cells.

**DOI:** 10.1038/bjc.1996.654

**Published:** 1996-12

**Authors:** L. Orlandi, N. Zaffaroni, A. Bearzatto, R. Silvestrini

**Affiliations:** Divisione di Oncologia Sperimentale C, Istituto Nazionale per lo Studio e la Cura dei Tumori, Milan, Italy.

## Abstract

**Images:**


British Journal of Cancer (1996) 74, 1924–1928
© 1996 Stockton Press All rights reserved 0007–0920/96 $12.00

### Effect of melphalan and hyperthermia on p34$^{cdc2}$ kinase activity in human melanoma cells

L Orlandi, N Zaffaroni, A Bearzatto and R Silvestrini

*Divisione di Oncologia Sperimentale C, Istituto Nazionale per lo Studio e la Cura dei Tumori, Via Venezian 1, 20133 Milan, Italy.*

**Summary** We previously reported that combined treatment with melphalan and mild hyperthermia (1 h at 42°C) caused a synergistic cytotoxic effect in JR8 melanoma cells, paralleled by a stabilisation of a melphalan-induced $G_2$-phase cell block. In this study, we investigated the effect of melphalan and hyperthermia on proteins that regulate $G_2$–M transition. Neither hyperthermia nor melphalan at a concentration of 2.5 $\mu$g ml$^{-1}$, which had no antiproliferative effect at 37°C, interfered with cyclin $B_1$ expression or p34$^{cdc2}$ kinase activity. At a concentration of 8.5 $\mu$g ml$^{-1}$, which reduced cell growth by 50% at 37°C, melphalan inhibited p34$^{cdc2}$ kinase activity as a consequence of an increased tyrosine phosphorylation of the protein. A similar inhibitory effect on p34$^{cdc2}$ kinase was obtained when the lowest melphalan concentration (2.5 $\mu$g ml$^{-1}$) was used under hyperthermic conditions. Our results indicate that thermal enhancement of melphalan cytotoxicity could be mediated at least in part by an inhibition of p34$^{cdc2}$ kinase activity, which prevents cell progression into mitosis.

**Keywords:** melphalan; hyperthermia; melanoma; $G_2$–M transition; p34$^{cdc2}$; cyclin $B_1$

Clinical studies on melanoma (Santinami *et al*., 1989) have shown that hyperthermia increases the anti-tumour activity of some bifunctional alkylating agents such as L-phenylalanine mustard (melphalan, L-PAM). The mechanisms responsible for the thermal enhancement of L-PAM activity are not yet completely understood. In experimental systems (1) an increase in L-PAM influx leads to a higher intracellular drug accumulation (Bates and MacKillop, 1989), (2) alteration of the DNA quaternary structure, which favours alkylation (Mills and Meyn, 1981), (3) interference with drug–DNA adduct metabolism (Zaffaroni *et al*., 1992) and (4) inhibition of DNA repair (Jorritsma *et al*., 1985) have been demonstrated. Moreover, we recently showed that hyperthermia can stabilise the transient accumulation of cells in $G_2$-phase induced by L-PAM in human melanoma cells (Orlandi *et al*., 1995).

Considerable progress has been made in understanding the proteins which regulate cell cycle progression. Specifically, the primary participants in $G_2$–M transition are a cyclin-dependent kinase, p34$^{cdc2}$, and the cyclin B protein, which regulates the activity of the kinase (Lewin, 1990; Nurse, 1990; Solomon *et al*., 1990). Here, we investigated the effect exerted by L-PAM, under normothermic and hyperthermic conditions, on the expression of cyclin $B_1$ protein as well as on the catalytic activity of p34$^{cdc2}$ kinase in a human melanoma cell line.

### Materials and methods

### *Cell line*

The human melanoma cell line JR8 (Zupi *et al*., 1985) was grown at 37°C in a 5% carbon dioxide humidified atmosphere in air, with RPMI-1640 medium (Bio Whittaker, Verviers, Belgium), containing 10% fetal calf serum (Biological Industries, Kibbutz Beth Haemek, Israel), 2 mM L-glutamine and gentamycin (0.1 mg ml$^{-1}$). During the phase of exponential growth, JR8 cells have a doubling time of about 24 h. All experiments were performed within the tenth passage after thawing.

### *Heat and drug treatments*

Cells were exposed to heat by placing T-25 flasks in a controlled precision water bath at 42°C (±0.05°C) for 1 h. For treatment with L-PAM (Sigma, St Louis, MO, USA) in normothermic or hyperthermic conditions, cells were exposed for 1 h to different drug concentrations or to the drug solvent (control samples). The temperature and exposure times were chosen because they correspond to the treatment perfusion in melanoma patients (Vaglini *et al*., 1986; Ghussen *et al*., 1988). Following treatment with hyperthermia, L-PAM, or both, cells were rinsed with phosphate-buffered saline (PBS) and fed with fresh medium. Each experimental point was run in triplicate. Experiments were repeated three times. Treatment-induced cytotoxicity was determined with a 96 h growth inhibition assay by using an electronic cell counter (Coulter Electronics, Hialeah, FL, USA) as previously described (Orlandi *et al*., 1995). Results were expressed as the cell number of treated samples compared with control samples.

### *Bivariate flow cytometric analysis of DNA content/cyclin $B_1$ expression*

At different intervals after treatment, cells were fixed in acetone and absolute ethanol (1:1) for 30 min at −20°C, then centrifuged and washed with 70% ethanol and PBS. The cells were incubated overnight with the monoclonal antibody to human cyclin $B_1$ (PharMingen, San Diego, CA, USA) at a dilution of 1:400 in PBS containing 1% bovine serum albumin (BSA), then washed and incubated with FITC-conjugated goat anti-mouse IgG antibody (Sigma) diluted 1:40 in PBS containing 1% BSA. The cells were resuspended in 10 $\mu$g ml$^{-1}$ propidium iodide and 0.1% RNAase in PBS and incubated at room temperature for 30 min in the dark (Gong *et al*., 1993). The fluorescence of stained cells was measured using a FACScan flow cytometer (Becton Dickenson, San José, CA, USA). Data were acquired and processed with Lysis II software (Becton Dickinson). A minimum of $10^4$ cells was measured for each sample. The percentage of cells in the $G_2$M cell phase was evaluated by the Cell Fit software according to the SOBR model (Becton Dickinson).

### *Immunoblotting*

At 48 and 72 h after treatment, cells were lysed on ice with a RIPA buffer (20 mM Tris pH 7.4, 150 mM sodium chloride,

Correspondence: R Silvestrini
Received 4 April 1996; revised 3 July 1996; accepted 8 July 1996

5 mM EDTA, 1% NP40, 50 mM sodium fluoride and protease inhibitors aprotinin, leupeptin, pepstatin at a concentration of 10 $\mu g\ ml^{-1}$, and 2 mM phenylmethylsulphonylfluoride). Each lysate was centrifuged at 15 000 *g* for 20 min, and the protein content of each supernatant was quantified by the Bio-Rad protein assay. Total cellular protein (50 $\mu g$) boiled in 2 × sodium dodecyl sulphate (SDS) gel loading buffer (250 mM Tris pH 6.8, 2% SDS, 30% glycerol, 10% 2-mercaptoethanol and 0.01% bromophenol blue) was separated on 12% SDS-polyacrylamide gel and transferred to nitrocellulose. Filters were blocked overnight in TBS-T buffer (20 mM Tris, 137 mM sodium chloride, pH 7.6, 0.1% Tween 20) with 5% skim milk and then incubated with the primary monoclonal antibody anti-p34[cdc2] (Santa Cruz Biotechnology, Santa Cruz, CA, USA). Unbound antibody was removed and filters were then incubated with the secondary antibody anti-mouse Ig horseradish peroxidase-linked whole antibody (Amersham, Bucks, UK). Bound antibody was detected using the enhanced chemiluminescence Western blotting detection system (Amersham). To reprobe with alternative antisera, the membranes were immersed in a stripping solution (100 mM 2-mercaptoethanol, 2% SDS, 62.5 mM Tris, pH 6.7) for 30 min at 50°C. Non-specific binding sites were blocked in 5% skim milk/TBS-T and the filter reprobed with anti-phosphotyrosine (Boehringer Mannheim, Mannheim, Germany) as primary antibody.

*Immunoprecipitation and histone $H_1$ kinase assay*

Total cellular protein (100 $\mu g$), obtained by lysing cells with RIPA buffer as described before, was immunoprecipitated by anti-p34[cdc2] agarose conjugate (Santa Cruz Biotechnology) for 4 h at 4°C. Immunoprecipitates were then washed four times with RIPA buffer and resuspended in 50 $\mu l$ of kinase buffer containing 50 mM Tris, pH 7.4, 10 mM magnesium chloride, 1 mM dithiothreitol and 50 $\mu g\ ml^{-1}$ histone $H_1$ (Boehringer Mannheim). Following a preincubation of 10 min at 30°C, reactions were started by the addition of 10 $\mu Ci$ of [$\gamma$-$^{32}P$]ATP (specific activity 3000 Ci $mmol^{-1}$), incubated at 30°C for 20 min, and stopped by the addition of 50 $\mu l$ of 2 × SDS gel loading buffer. The mixtures were denatured at 95°C for 5 min and separated on 12% SDS-polyacrylamide gel. Bands were detected by autoradiography and quantified by an Ultrascan XL enhanced laser densitometer (LKB, Turku, Finland).

**Table I** $G_2M$ cell fraction and cyclin $B_1$-expressing cells at different intervals after treatment

| | *Control (37°C)* | *L-PAM 37°C 2.5 μg ml⁻¹* | *L-PAM 37°C 8.5 μg ml⁻¹* | *Control (42°C)* | *L-PAM 42°C 2.5 μg ml⁻¹* |
|---|---|---|---|---|---|
| 0 h | | | | | |
| $G_2M$ | 12+3 | 15+2 | 13+4 | 15+3 | 17+4 |
| Cyclin $B_1$ | 18±4 | 18±4 | 20±5 | 18±2 | 20±7 |
| 24 h | | | | | |
| $G_2M$ | 13±6 | 13±4 | 13±2 | 14±5 | 15±5 |
| Cyclin $B_1$ | 19±5 | 23±5 | 24±4 | 18±6 | 26±8 |
| 48 h | | | | | |
| $G_2M$ | 15±3 | 24±5 | 27±4[a] | 12±5 | 34±3[a] |
| Cyclin $B_1$ | 17±4 | 29±7 | 35±3[a] | 19±3 | 40±4[a] |
| 72 h | | | | | |
| $G_2M$ | 13±2 | 15±4 | 62±5[a] | 10±4 | 59±5[a] |
| Cyclin $B_1$ | 18±4 | 22±3 | 60±7[a] | 16±3 | 63±5[a] |
| 96 h | | | | | |
| $G_2M$ | 10±5 | 11±6 | 33±5[a] | 9±4 | 43±4[a] |
| Cyclin $B_1$ | 11±3 | 15±7 | 35±7[a] | 13±5 | 45±7[a] |

Data represent mean values±s.d. from three independent experiments. [a]$P<0.05$, Student's *t*-test, compared with control at 37°C.

### Results

*Effect of hyperthermia and/or L-PAM on tumour cell growth*

A 1 h exposure to 42°C did not induce any appreciable effect in JR8 cell growth at 96 h after treatment. Specifically, a very modest inhibition (−5%) was observed in the cell number of heat-treated cells as compared with controls. In normothermic conditions, a 1 h exposure to L-PAM produced a negligible decrease (−6%) in cell proliferation at the lowest concentration of 2.5 $\mu g\ ml^{-1}$. The growth inhibitory effect was considerably higher (−50%) when cells were exposed to the highest drug concentration of 8.5 $\mu g\ ml^{-1}$. A similar antiproliferative effect (−46%) was reached after combined treatment with 2.5 $\mu g\ ml^{-1}$ L-PAM under hyperthermic conditions.

**Figure 1** Variation in the percentage of $G_2M$ cells (**a**) and cyclin $B_1$-expressing cells (**b**) at different intervals after a 1 h exposure to L-PAM and/or hyperthermia. Results were obtained by bivariate flow cytometric analysis of DNA content/cyclin $B_1$ expression, as described in Materials and methods. Control cells maintained at 37°C without any treatment (○—○); cells exposed to 42°C (○- - - ○), 2.5 $\mu g\ ml^{-1}$ L-PAM at 37°C (●—●), 8.5 $\mu g\ ml^{-1}$ L-PAM at 37°C (■–■) and 2.5 $\mu g\ ml^{-1}$ L-PAM at 42°C (●- - -●).

*Effect of hyperthermia and/or L-PAM on $G_2M$ cell fraction and cyclin $B_1$ expression*

Hyperthermia did not induce any variation in the $G_2M$ cell fraction (Table I and Figure 1a). Exposure to the lowest L-PAM concentration (2.5 $\mu g\ ml^{-1}$) produced a slight and temporary increase in the $G_2M$ cell fraction, appreciable only at 48 h. Following exposure to the highest L-PAM concentration (8.5 $\mu g\ ml^{-1}$), a persistent block of cells in the $G_2M$-phase was observed. Such an accumulation was maximum at 72 h and, although to a lesser extent, still appreciable at 96 h. A comparable persistent $G_2M$ accumulation was obtained after treatment of cells with 2.5 $\mu g\ ml^{-1}$ under hyperthermic conditions. Such $G_2M$ accumulations were primarily caused by a block of cells in the $G_2$-phase, since the mitotic cells never accounted for more than 2% of the overall cell population (data not shown).

The fraction of cyclin $B_1$-expressing cells, determined by flow cytometry on the same cell samples, was similar to the fraction of $G_2M$-phase cells in control samples as well as in samples treated with L-PAM, hyperthermia, or both (Table I and Figure 1b).

*Effect of hyperthermia and/or L-PAM on the catalytic activity of $p34^{cdc2}$ kinase*

At 48 h and 72 h after treatment, the JR8 cell extract was immunoprecipitated by using anti-$p34^{cdc2}$ antibody and the kinase activity measured by using histone $H_1$ as a substrate (Figure 2). Immunoprecipitates obtained from cells treated with hyperthermia or with 2.5 $\mu g\ ml^{-1}$ L-PAM phosphorylated histone $H_1$ to a similar degree as in control cells (Table II). Conversely, immunoprecipitates obtained 48 h and 72 h after treatment with 8.5 $\mu g\ ml^{-1}$ L-PAM showed a strongly decreased kinase activity compared with controls. A similarly pronounced inhibition of $p34^{cdc2}$ catalytic activity was seen in immunoprecipitates obtained from cells exposed to the combined treatment with 2.5 $\mu g\ ml^{-1}$ L-PAM and hyperthermia.

**Figure 2** A representative experiment illustrating the effect of a 1 h exposure to L-PAM and/or hyperthermia on the catalytic activity of $p34^{cdc2}$ kinase in the JR8 cell line. An aliquot of 100 $\mu g$ of total cell proteins obtained from cells 48 h and 72 h after treatment was immunoprecipitated with anti-$p34^{cdc2}$ monoclonal antibody, and histone $H_1$ kinase activity of immunoprecipitates (IP) was analysed as described in Materials and methods. Lane 1, control cells (maintained at 37°C without any treatment); lane 2, cells treated with 2.5 $\mu g\ ml^{-1}$ L-PAM at 37°C; lane 3, cells treated with 8.5 $\mu g\ ml^{-1}$ L-PAM at 37°C; lane 4, cells exposed to 42°C; lane 5, cells treated with 2.5 $\mu g\ ml^{-1}$ L-PAM at 42°C.

*Effect of hyperthermia and/or L-PAM on the expression and phosphorylation state of $p34^{cdc2}$*

To investigate whether inhibition of the kinase catalytic activity was ascribable to a decreased expression of $p34^{cdc2}$, we examined the protein levels at 48 h and 72 h after exposure of cells to hyperthermia, L-PAM, or both. No remarkable difference was observed in the level of $p34^{cdc2}$ among the different treatment groups (Figure 3, upper panel and Table II). Since $p34^{cdc2}$ catalytic activity is known to be modulated by phosphorylation of its tyrosine and threonine residues, we also investigated the effect of hyperthermia and L-PAM, singly or in association, on the phosphorylation state of $p34^{cdc2}$. The immunoblots with anti-phosphotyrosine antibody (Figure 3, lower panel and Table II) showed that samples treated with hyperthermia alone or with 2.5 $\mu g\ ml^{-1}$ L-PAM exhibited a $p34^{cdc2}$ tyrosine phosphorylation similar to that of control samples. Conversely, samples exposed to 8.5 $\mu g\ ml^{-1}$ L-PAM showed an increase in the degree of tyrosine phosphorylation. A similarly pronounced increase was observed in samples exposed to the combined treatment with 2.5 $\mu g\ ml^{-1}$ L-PAM and hyperthermia.

### Discussion

We investigated the effect of hyperthermia and L-PAM on $G_2-M$ transition in a human melanoma cell line. A 1 h exposure to 42°C did not affect the growth of JR8 cells. Such a result is in agreement with previous evidence by our (Zaffaroni *et al.*, 1992; Orlandi *et al.*, 1993, 1995) and other groups (Rofstad *et al.*, 1990), indicating a negligible effect of mild hyperthermia itself on human melanoma-established cell lines and primary cultures. Moreover, clinically mild hyperthermia is devoid of any significant anti-tumour effect, and it is generally used in conjunction with alkylating agents to modulate drug activity positively in drug-refractory tumours, such as melanoma (Santinami *et al.*, 1989; Ghussen *et al.*, 1988). In JR8 cells, hyperthermia markedly increased the cytotoxic effect of a low L-PAM concentration and stabilised the transient L-PAM-induced $G_2$ accumulation. Under normothermic conditions, such a low L-PAM concentration only induced a negligible inhibitory effect on cell growth and a modest increase in $G_2$ cell fraction,

**Table II** Densitometric analysis of the immunoblotting or histone $H_1$ kinase activity

| | 37°C | | | 42°C | |
|---|---|---|---|---|---|
| | | *L-PAM* | | | *L-PAM* |
| | *Control* | *2.5 $\mu g\ ml^{-1}$* | *8.5 $\mu g\ ml^{-1}$* | *Control* | *2.5 $\mu g\ ml^{-1}$* |
| At 48 h | | | | | |
| Immunoblotting | | | | | |
| $p34^{cdc2}$ | 1.00 | $0.93 \pm 0.15$ | $1.13 \pm 0.11$ | $0.95 \pm 0.13$ | $1.10 \pm 0.11$ |
| Phosphotyrosine | 1.00 | $0.96 \pm 0.13$ | $1.30 \pm 0.14$ | $0.89 \pm 0.10$ | $1.45 \pm 0.18^a$ |
| Kinase activity | 1.00 | $1.15 \pm 0.13$ | $0.35 \pm 0.15^a$ | $0.95 \pm 0.07$ | $0.17 \pm 0.07^a$ |
| At 72 h | | | | | |
| Immunoblotting | | | | | |
| $p34^{cdc2}$ | | $0.91 \pm 0.28$ | $0.82 \pm 0.25$ | $0.92 \pm 0.20$ | $0.89 \pm 0.14$ |
| Phosphotyrosine | | $0.95 \pm 0.14$ | $1.37 \pm 0.12$ | $0.96 \pm 0.15$ | $1.34 \pm 0.21$ |
| Kinase activity | | $1.07 \pm 0.21$ | $0.28 \pm 0.10^a$ | $0.94 \pm 0.08$ | $0.19 \pm 0.08^a$ |

The relative intensity of the bands of the immunoblot analysis performed with anti-$p34^{cdc2}$ or anti-phosphotyrosine monoclonal antibodies and of kinase assay are reported. Exent of the signal was quantified by using an ultrascan XL densitometer. Data are shown as the ratio to the control at 37°C and represent mean values $\pm$s.d. from three independent experiments. $^aP < 0.05$, Student's *t*-test, compared with control at 37°C.

**Figure 3** A representative experiment illustrating the effect of a 1 h exposure to L-PAM and/or hyperthermia on the expression and phosphorylation state of p34$^{cdc2}$ kinase in the JR8 cell line. Cells were harvested at 48 h and 72 h after treatment and processed by immunoblot analysis. Whole cell extract (50 $\mu$g) was separated and electrophoretically blotted. Proteins were probed with anti-p34$^{cdc2}$ (upper panel) and reprobed, after filter stripping, with anti-phosphotyrosine (lower panel). Lane 1, control cells (maintained at 37°C without any treatment); lane 2, cells treated with 2.5 $\mu$g ml$^{-1}$ L-PAM at 37°C; lane 3, cells treated with 8.5 $\mu$g ml$^{-1}$ L-PAM at 37°C; lane 4, cells exposed to 42°C; lane 5, cells treated with 2.5 $\mu$g ml$^{-1}$ L-PAM at 42°C.

appreciable 48 h after treatment. Cytotoxic effects and cell cycle perturbations comparable to those produced by combined treatment were obtained with L-PAM under normothermic conditions using a more than 3-fold drug concentration.

The mechanism by which hyperthermia can prolong the $G_2$ cell block induced by L-PAM has not yet been investigated. In DNA lesions, L-PAM produces interstrand cross-links, which may arrest cells in $G_2$ by preventing strand segregation. If this were true, it could be that persistence of $G_2$ accumulation depends on the stability of DNA cross-links (Konopa, 1988). In a previous study on melanoma primary cultures, we demonstrated that hyperthermia was able to increase the accumulation of L-PAM-induced DNA interstrand cross-links and to prevent their long-term removal (Zaffaroni *et al*., 1992). However, the coupling of DNA damage to cell cycle perturbation induced by L-PAM is still largely unclear.

Hyperthermia could also stabilise L-PAM-induced $G_2$ accumulation through an inhibitory effect on proteins that regulate $G_2-M$ transition. Such a transition in eukaryotic cells is controlled by a mitosis-promoting factor (MPF), which consists of a regulatory subunit, a specific B-type cyclin, and the catalytic subunit, p34$^{cdc2}$ kinase (Lewin, 1990; Nurse, 1990; Norbury and Nurse, 1992).

Results we obtained from DNA/cyclin $B_1$ bivariate flow cytometric analysis performed on JR8 cells exposed to L-PAM under normothermic or hyperthermic conditions indicated that hyperthermia, L-PAM or both did not reduce the expression of cyclin $B_1$, thus suggesting that this regulatory protein is not an important target for L-PAM/hyperthermia-induced cell cycle arrest. Conversely, the highest concentration of L-PAM, under normothermic conditions, and the lowest one, in conjunction with hyperthermia, induced a marked inhibition of p34$^{cdc2}$ kinase activity in correspondence with the accumulation of cells in the $G_2$-phase. Such a negative modulation of p34$^{cdc2}$ kinase activity was not caused by an alteration in the overall level of the protein. This finding, together with the evidence that cyclin $B_1$ protein level continued to accumulate after L-PAM/hyperthermia treatment, suggested that cell cycle delay is not mediated through a lack of MPF complex formation.

Exposure to the highest L-PAM concentration at 37°C, as well as to the lowest one at 42°C, enhanced the level of tyrosine phosphorylation of p34$^{cdc2}$. Such an effect was more pronounced after combined L-PAM/hyperthermia treatment. Since activation of MPF results from the dephosphorylation of cyclin B-bound p34$^{cdc2}$, our findings suggest that a treatment-induced $G_2$ accumulation results from the inability to activate MPF. The lack of MPF activity may be due to an up-regulation of cdc2 tyrosine kinase activity or a down-regulation of tyrosine phosphatase activity. Recent data (O'Connor *et al*., 1992) indicated that inhibition of p34$^{cdc2}$ kinase activity by another bifunctional alkylating agent, nitrogen mustard ($HN_2$), on lymphoma cells was consequent on a down-regulation of the cdc25 tyrosine phosphatase (O'Connor *et al*., 1994).

On the whole, our results indicate thermal stabilisation of cell cycle perturbations induced by L-PAM as one of the possible mechanisms for the enhancement of L-PAM cytotoxic activity under hyperthermic conditions. Specifically, hyperthermia seems to enhance the inhibition of p34$^{cdc2}$ kinase activity induced by L-PAM as a consequence of an increased tyrosine phosphorylation of the protein.

**Acknowledgements**
This project was supported by grants from the Italian Health Ministry and the Italian Association for Cancer Research (AIRC). The authors thank E Ronchi for densitometric analysis, B Johnston and B Canova for editing and typing the manuscript.

### References

BATES DA AND MACKILLOP WJ. (1989). Effect of hyperthermia on the uptake and cytotoxicity of melphalan in Chinese hamster ovary cells. *Int. J. Radiat. Oncol. Biol. Phys*., **16**, 187–191.

GONG J, TRAGANOS F AND DARZYNKIEWICZ Z. (1993). Simultaneous analysis of cell cycle kinetics at two different DNA ploidy levels based on DNA content and cyclin B measurements. *Cancer Res*., **53**, 5096–5099.

GHUSSEN F, KRUGER I AND GROTH W. (1988). The role of regional hyperthermic cytostatic perfusion in the treatment of extremity melanoma. *Cancer*, **61**, 654–658.

JORRITSMA JB, KAMPINGA HH, SCAF AH AND KONINGS AW. (1985). Strand break repair, DNA polymerase activity and heat radiosensitization in thermotolerant cells. *Int. J. Hyperthermia*, **1**, 131–145.

KONOPA J. (1988). $G_2$ block induced by DNA crosslinking agents and its possible consequences. *Biochem. Pharmacol*., **37**, 2303–2309.

LEWIN B. (1990). Driving the cell cycle: M phase kinase, its partners, and substrates. *Cell*, **61**, 743–752.

MILLS MD AND MEYN RE. (1981). Effects of hyperthermia on repair of radiation-induced DNA strand breaks. *Radiat. Res*., **87**, 314–328.

NORBURY C AND NURSE P. (1992). Animal cell cycles and their control. *Annu. Rev. Biochem*., **61**, 441–470.

NURSE P. (1990). Universal control mechanism regulating onset of M-phase. *Nature*, **344**, 503–508.

O'CONNOR PM, FERRIS DK, WHITE GA, PINES J, HUNTER T, LONGO DL AND KOHN KW. (1992). Relationships between cdc2 kinase, DNA cross-linking, and cell cycle perturbations induced by nitrogen mustard. *Cell Growth Diff*., **3**, 43–52.

O'CONNOR PM, FERRIS DK, HOFFMANN I, JACKMAN J, DRAETTA G AND KOHN KW. (1994). Role of the cdc25C phosphatase in $G_2$ arrest induced by nitrogen mustard. *Proc. Natl. Acad. Sci. USA*, **91**, 9480–9484.

ORLANDI L, COSTA A, ZAFFARONI N, VILLA R, VAGLINI M AND SILVESTRINI R. (1993). Relevance of cell kinetics and ploidy characteristics for the thermal response of malignant melanoma primary cultures. *Int. J. Oncol*., **2**, 523–526.

ORLANDI L, ZAFFARONI N, BEARZATTO A, COSTA A, SUPINO R, VAGLINI M AND SILVESTRINI R. (1995). Effect of melphalan and hyperthermia on cell cycle progression and cyclin $B_1$ expression in human melanoma cells. *Cell Prolif.*, **28,** 617–630.

ROFSTAD EK, ZAFFARONI N AND HYSTAD ME. (1990). Heterogeneous radiation and heat sensitivity *in vitro* of human melanoma xenograft lines established from different lesions in the same patient. *Int. J. Radiat. Biol.*, **57,** 1113–1122.

SANTINAMI M, BELLI F, CASCINELLI N, ROVINI D AND VAGLINI M. (1989). Seven years experience with hyperthermic perfusions in extracorporeal circulation for melanoma of the extremities. *J. Surg. Oncol.*, **42,** 201–208.

SOLOMON MJ, GLOTZER N, LEE TH, PHILIPPE M AND KIRSCHNER MW. (1990). Cyclin activation of p34$^{cdc2}$. *Cell*, **63,** 1013–1024.

VAGLINI M, SANTINAMI M, NAVA M. (1986). Hyperthermic antiblastic perfusion in extracorporeal circulation: surgical technique and results in the treatment of extremities tumors. *J. Extra-Corpor. Technol.*, **18,** 13–21.

ZAFFARONI N, VILLA R, ORLANDI L, VAGLINI M AND SILVESTRINI R. (1992). Effect of hyperthermia on the formation and removal of DNA interstrand cross-links induced by melphalan in primary cultures of human malignant melanoma. *Int. J. Hyperthermia*, **8,** 341–349.

ZUPI G, MAURO F, BALDUZZI MA, PARDINI MC, CAVALIERE R AND GRECO C. (1985). Established melanoma cell lines from different metastatic nodules of a single patient. A useful model for cancer therapy. *Proc. Am. Assoc. Cancer Res.*, **26,** 22.